# Principles of Spatial Transcriptomics Analysis: A Practical Walk-Through in Kidney Tissue

**DOI:** 10.3389/fphys.2021.809346

**Published:** 2022-01-06

**Authors:** Teia Noel, Qingbo S. Wang, Anna Greka, Jamie L. Marshall

**Affiliations:** ^1^Kidney Disease Initiative, Broad Institute of MIT and Harvard, Cambridge, MA, United States; ^2^Program in Medical and Population Genetics, Broad Institute of MIT and Harvard, Cambridge, MA, United States; ^3^Program in Bioinformatics and Integrative Genomics, Harvard Medical School, Boston, MA, United States; ^4^Analytic and Translational Genetics Unit, Massachusetts General Hospital, Boston, MA, United States; ^5^Department of Statistical Genetics, Graduate School of Medicine, Osaka University, Osaka, Japan; ^6^Department of Medicine, Brigham and Women’s Hospital, Harvard Medical School, Boston, MA, United States

**Keywords:** slide-seqV2, spatial transcriptomics, kidney spatial transcriptomics, slide-seq, kidney transcriptomics

## Abstract

Spatial transcriptomic technologies capture genome-wide readouts across biological tissue space. Moreover, recent advances in this technology, including Slide-seqV2, have achieved spatial transcriptomic data collection at a near-single cell resolution. To-date, a repertoire of computational tools has been developed to discern cell type classes given the transcriptomic profiles of tissue coordinates. Upon applying these tools, we can explore the spatial patterns of distinct cell types and characterize how genes are spatially expressed within different cell type contexts. The kidney is one organ whose function relies upon spatially defined structures consisting of distinct cellular makeup. Thus, the application of Slide-seqV2 to kidney tissue has enabled us to elucidate spatially characteristic cellular and genetic profiles at a scale that remains largely unexplored. Here, we review spatial transcriptomic technologies, as well as computational approaches for cell type mapping and spatial cell type and transcriptomic characterizations. We take kidney tissue as an example to demonstrate how the technologies are applied, while considering the nuances of this architecturally complex tissue.

## Applications of Spatial Transcriptomics

Unbiased spatial transcriptomics adds *in situ* spatial context to single cell RNA data providing a powerful tool to characterize the spatial location for whole transcriptome sequencing (10x Genomics, 2007; Chen et al., 2015; Stegle et al., 2015; Ståhl et al., 2016; Rodriques et al., 2019; Liao et al., 2021; Longo et al., 2021; Nature Methods, 2021; Stickels et al., 2021). This is accomplished with two technologies, Spatial Transcriptomics (ST or Visium) and Slide-seqV2 (10x Genomics, 2007; Ståhl et al., 2016; Rodriques et al., 2019; Stickels et al., 2021). Other technologies, such as MERFISH (targeted), GeoMx™ (targeted), DBiT-seq, and Stereo-seq (BGI), allow for higher resolution, in some cases even subcellular, detection of RNA (Chen et al., 2015, 2021; Liu et al., 2020; Zollinger et al., 2020; Su et al., 2021). However, they tend to be more complex to implement. Both ST and Slide-seqV2 use uniquely barcoded beads with Oligo(dT) to capture polyadenylated RNA. ST has a larger feature size (avg 1–10 cells per spot, 50 μm beads with 100 μm spacing between beads (10x Genomics, 2007) than Slide-seqV2 [avg 1–3 cells per spot, 10 μm beads (Rodriques et al., 2019; Stickels et al., 2021)] and thus has relatively limited resolution compared to the near single cell resolution in Slide-seqV2. On the other hand, ST allows for profiling of a large area of tissue (typically the entire cross section) while a single Slide-seqV2 array only covers a 3 mm diameter of tissue so multiple arrays on serial cryosections are needed to cover the entire tissue cross section. The other advantage of ST is the ability to co-stain the same tissue slice from which the spatial transcriptome is captured with hematoxylin and eosin staining (H&E) for histology or targeted antibodies. Slide-seqV2 and ST capture similar numbers of UMIs across the same spot area (Stickels et al., 2021).

Human and mouse kidneys across health and disease have been profiled with both ST and Slide-seqV2 (Raghubar et al., 2020; Lake et al., 2021; Melo Ferreira et al., 2021). Raghubar et al. (2020) used ST to examine spatial transcriptomic differences between sex and species of human and mouse kidney tissue revealing differences in gene expression correlating with male versus female kidneys and human versus mouse kidneys. Raghubar et al. (2020) used ST to characterize spatial transcriptomics and in particular immune cell clusters in human kidney tissue and mouse models of kidney tissue subjected to ischemia/reperfusion injury and cecal ligation puncture (Melo Ferreira et al., 2021). Raghubar et al. (2020) used both ST and Slide-seqV2 to spatially define altered injury states and a fibrotic niche in human kidney biopsies from healthy, acute kidney injury (AKI), and chronic kidney disease (CKD) participants (Lake et al., 2021).

Our group used Slide-seqV2 to develop a spatial transcriptomic atlas of human and mouse kidney tissue in health and disease (Marshall et al., 2021). We profiled two mouse models of disease, early diabetic kidney disease (DKD, BTBR *ob/ob*) and uromodulin autosomal dominant tubulointerstitial kidney disease (ADTKD, UMOD-KI). The study also contains nine human participants with both cortex and medulla biopsies, one with early DKD and one with injury due to sustained tumor compression. Slide-seqV2 revealed the spatial location of *LYVE1*+ macrophages in human medulla with injury due to sustained tumor compression. In mice with early DKD, we revealed changes in the cellular organization of spatially restricted glomeruli. In UMOD-KI mice, we identified the spatial locations of diseased fibroblasts, macrophages, and *Trem2* + macrophages as well as an upregulation of the unfolded protein response (UPR) pathway in thick ascending limb (TAL) tubules. These results altogether demonstrated the utility of spatial transcriptomics technologies combined with downstream computational analysis to uncover previously unknown human and mouse kidney physiology. Throughout this review, we will discuss such computational approaches for cell type mapping as well as spatial cell type and transcriptomic characterizations focusing on kidney tissue.

## Preprocessing of Spatial Transcriptomics Data

The Slide-seqV2 reads are first aligned and mapped to the human or mouse reference transcriptome using tools such as STAR aligner (Dobin et al., 2013). After standard quality control protocols, including filtering based on the number of genes and UMIs per bead, the Slide-seqV2 data is turned into an expression matrix where rows correspond to beads and columns to genes. Additionally, per bead, native spatial coordinates are preserved across 2-D tissue space.

## Mapping the Cell Type From scRNA-Seq Data

The first step in the analysis of Slide-seqV2 data is to assign cell type identities to each bead. Accurate cell type classification is aided by external scRNA-seq datasets from published materials, where cell types are already annotated (Halpern et al., 2017; Lake et al., 2021; Subramanian et al., 2021). Different computational techniques have been developed to perform such analysis. First, we will focus on a method that was originally developed (Rodriques et al., 2019), NMFreg. Since typical scRNA-seq data is high dimensional (e.g., >10 thousands cells ×>20 thousands genes, where each cell is annotated with specific known cell type), the scRNA-seq expression matrix is projected to a low-dimensional basis of factors by selection of highly variable genes followed by NMF (Lee and Seung, 1999). Choosing the number of dimensions of the low-dimensional space is not trivial; In the Slide-seq paper (Rodriques et al., 2019), the authors evaluated different numbers of dimensions *k* to semi-manually assign a value to the parameter (they showed that the biological interpretation is roughly consistent across different *k*s). Every factor is then assigned a unique cell type. To do so, for every cell in the scRNA-seq data, the method computes a loading for all factors, and assigns the cell the factor of maximum weight. Since each cell was previously annotated with a cell type identity, the cell type distribution for the cells assigned to each factor can be calculated. Every factor is then assigned the cell type of maximum count.

Having acquired a set of “metagene” factors for each cell type category, the method next utilizes this set of features to map Slide-seqV2 beads to cell types. Each bead’s gene expression profile is approximated as a non-negative sum of the factors using non-negative least square regression (NNLS). Since each factor can be mapped to a unique cell type, the factor loading for each bead can be converted to cell type loading. The cell type with highest load is selected to define the cell type identity for each bead, and the beads that do not have a single dimension with clearly highest load (e.g., beads having uniform load over all the dimensions, or having >1 dimensions with ≫0 load) are filtered out. More recent developments of spatial transcriptomics cell type mapping techniques will be discussed in the next chapter.

## Cell Type Identification With Seurat Transfer Learning

**FIGURE 1:**
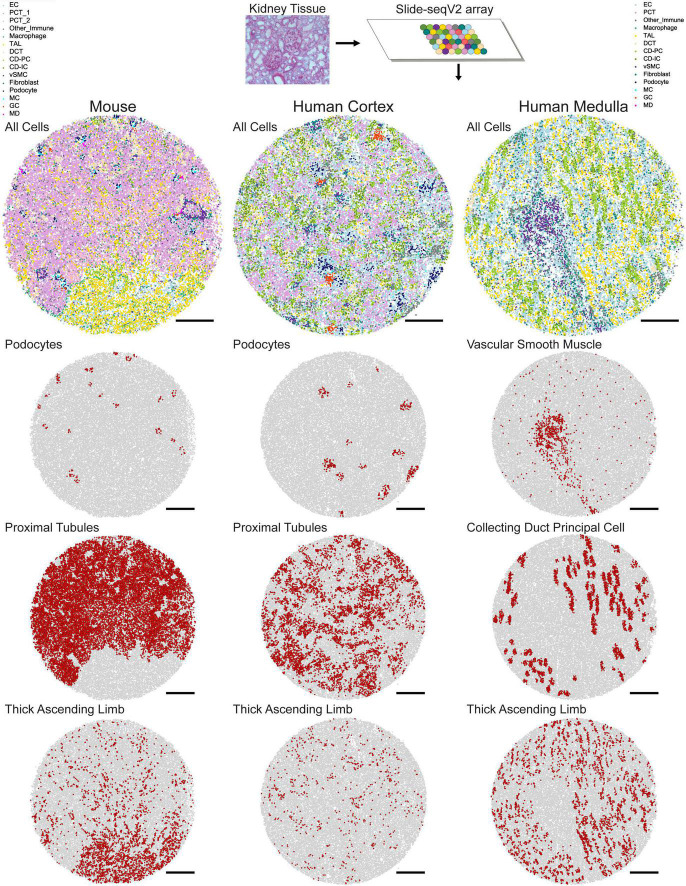
Mapped cell types in human and mouse kidney tissue. In Slide-seqV2, a 10 μm cryosection of kidney tissue is melted onto an array containing 10 μm beads which bind to messenger RNA. Once library preparation is complete, spatially barcoded cDNA corresponding to each bead is assigned a cell identity using Seurat transfer learning. Example mouse, human cortex, and human medulla tissue with all cell types mapped are shown. Individual mappings of podocytes, proximal tubules, and thick ascending limbs are shown for the mouse and human cortex arrays, while vascular smooth muscle, collecting duct principal cell, and thick ascending limb are shown for the human medulla array. Scale bars = 500 μm.

A second approach for assigning cell type identities to beads is Seurat’s transfer learning method (Stuart et al., 2019). Similar to NMFreg, an scRNA-seq data set is utilized to elucidate cell type labels in a spatial transcriptomics query data set. First, the data of both the reference and the query are projected to a shared lower-dimensional space. Anchors are then defined to map pairs of similar data points between the reference and the query, based on the amount of shared overlap between their respective neighborhoods. Next, weights are allocated to each anchor in order to emphasize mappings between similar biological states. The weights are based on the following criteria: (1) The distance between the query bead and k nearest reference neighbors and (2) The number of mutual nearest neighbors between the query bead and reference cell. Finally, cell type annotations from scRNA-seq are transferred along these anchors to the spatial transcriptomics reference using a weighted voter classifier (Kuncheva and Rodríguez, 2014). As a result of either the NMFreg or Seurat transfer learning methodologies, not only is each bead informed by a gene expression profile and spatial location, but also its overarching cell type identity. In Marshall et al. (2021), Seurat transfer learning presented higher consistency with physiologically known structures than NMFreg for all cell types in human tissue and in collecting ducts (CD), vascular smooth muscle cells (vSMC), and distal tubules in mouse tissue. An overview of mapped cell types in mouse and human tissue is shown in Figure 1.

## Decomposing Spatial Transcriptomic Spots Into Multiple Cell Types

One caveat of many spatial transcriptomics methods, including Slide-seqV2, is that data points are not necessarily representative of single cells. Even in near single-cell resolution spatial transcriptomic technologies such as Slide-seqV2, while data points are about the size of a single cell, their fixed locations may overlap with and capture mRNA from multiple cells. This is particularly apparent when spatial transcriptomics technologies are applied to kidney tissue, wherein the cell type landscape is dense and complex (Chabardès-Garonne et al., 2003; Raghubar et al., 2020). As a result, one collective shortcoming of methods like NMFreg and Seurat transfer learning is that they assume a one-to-one relationship between cell types and data points. More recent approaches have tried to dissect the multifaceted cell type profile of spatial transcriptomics spots, leading to a higher-resolution understanding of cell type distributions across tissue space. One method is RCTD, or robust cell type decomposition (Cable et al., 2021). RCTD leverages a generalized linear model for the total counts per gene on each data point. Specifically, counts are assumed to be Poisson distributed with the rate parameter being the product of the total molecules corresponding to a query data point and a mixture metric representative of the contribution of each gene to all cell types. In particular, one component of the mixture metric is the weighted sum of the average gene expression profiles per cell type (derived from a scRNA-seq reference dataset). One notable asset of RCTD is that it explicitly incorporates into its model platform effects that may mask true biological signals. Ultimately the weights that best fit the observed gene counts in a query data point indicate the proportions of each cell type captured by this data point.

A second example of a method that is able to decompose spatial transcriptomic spots into multiple cell types is SPOTLight (Elosua-Bayes et al., 2021). SPOTLight follows the same trajectory as NMFreg, using both NMF and NNLS at its essence, but incorporates a more biologically driven initialization step and additional NNLS step. First, SPOTLight learns a *k*-dimensional set of factors (*k* = number of cell type categories) from a scRNA-seq reference *via* NMF. While the initial factorization may be chosen to be random, here, the authors chose to initialize the matrices so that they encode gene markers for each cell type in the first matrix, and cell type identities of cells in the second matrix. NMF is then run to find the factorization of genes by factors and factors by cells that best fits the scRNA-seq reference. An initial round of NNLS derives the loadings of the basis of factors defined in the previous step for every spatial transcriptomics spot. A second round of NNLS formalizes the relationship between spots and cell types as follows: First, it defines a matrix of factors by cell types, where every column represents the median factor profile per cell type (derived from the first NMF step). Second, the spatial transcriptomic spots are decomposed into a weighted combination of these cell type profiles.

In comparison to RCTD, SPOTlight does not account for platform effects. On the other hand, RCTD has two modes: doublet, which assumes a maximum of two cell types per data point and full, which allows for >2 cell types. The authors forewarn that performance is largely dictated by the mode that the user opts for; in particular, full mode may be subject to overfitting the data. In the context of the kidney, which is complex and tightly structured, doublet mode is not a safe assumption to make. On the other hand, SPOTlight does not make this distinction and searches for the maximum number of cell types, and its proven accuracy is based on this framework.

Additional notable methods have been developed to increase our confidence of both singular and multiple cell type calls per spot. Confidence in cell type classifications can be hindered by the sparse UMI-capture rate of spatial transcriptomic spots in comparison to scRNA-seq. FIST addresses this issue by imputing transcriptomes based on spatial relationships of data points (Li Z. et al., 2021). SPICEMIX utilizes both intrinsic transcriptomic information and the spatial relationships of data points to infer cell type identities (Chidester et al., 2021). Cell2Location utilizes a Bayesian model to predict cell type identities (Kleshchevnikov et al., 2020). Lastly, CellDART tackles the problem of identifying the cell type composition of each spot by leveraging neural networks (Bae et al., 2021).

## Spatial Curation of Cell Type Identities

The cell type identities assigned from the observed gene expression markers through computational techniques is a noisy estimate. For example, the set of mRNAs detected in a 2-D plane might not be representative of the global distribution. As a result, it is advantageous to have prior knowledge of the spatial distribution of cell types across cross-sectional tissue space as a method of further filtering out noisy cell type calls. For example, we know that podocytes occur solely within circular structures called glomeruli (Garg, 2018), and distal convoluted tubules (DCT) and principal cells of the collecting duct (CD-PC) form tubular structures (McCormick and Ellison, 2015). In our work, we have implemented automated methods to identify and filter out isolated podocyte or distal tubule calls that do not occur within these denser structures. Specifically, cell type calls that have less than *k* nearest neighbors with the cell type identity of interest are filtered out (Marshall et al., 2021).

Furthermore, we can use prior knowledge about the known vicinity of multiple cell type classes to hone in on biologically sensical cell type calls. For example, granular cells, macula densa, and glomeruli are known to be adjacent to each other in a structure called the juxtaglomerular apparatus (JGA)(Briggs and Schnermann, 1996). The polygon encapsulating each of these structures can be found and a filtration system can be set up where only those within *x* units of each other are maintained. Similarly, intercalated cells of the collecting duct (CD-IC) occur within larger tubular structures made up of CD-PC (Rao et al., 2019). Again, we can isolate instances of CD-IC that occur within *x* units of the edge of any CD-PC structure.

Lastly, certain cell types are thought to occur only within certain regions of tissue. In the kidney, two such regions are the cortex and the medulla. Our prior knowledge (Kriz and Kaissling, 2008) suggests that cell types belonging to glomerular structures (i.e., podocytes, mesangial cells, and endothelial cells) and proximal tubule cells occur only within the cortex. On the other hand, DCT, CD-IC, CD-PC, and the thick ascending limb (TAL) are enriched and form dense structures within the medulla. We can use our knowledge of these distributions of cells across the cortex and medulla to delineate the two regions, and further filter cell types that are called in the unlikely zones.

Not only are cell type calls trusted based on the agreement between their spatial distribution and known morphology of kidney tissue, but we can also verify their identities by looking at the relative expression of known cell type marker genes. Furthermore, cell type proportions can be computed and compared between Slide-seqV2 arrays and Hybridization Chain Reaction (HCR) images (Choi et al., 2018; Marshall et al., 2021).

## Downstream Analyses

Once cell type identities are well-defined across spatial transcriptomic data, the spatial distribution of data points can be explored at two levels: (1) cell types and (2) gene expression profiles within certain cell types. In terms of the former, we can simply ask what the cell type composition looks like in a tissue section at large, or in the medulla or cortex regions. Furthermore, we may be interested in the neighborhoods of certain cell types. That is, what cell types often neighbor a cell type of interest. In our work, we target cell type neighborhoods of *Trem2*+ and *LYVE1*+ macrophages using *k* nearest neighbors (Marshall et al., 2021). Lastly, we can look at the morphology of cell type structures. Utilizing computational geometry (Gillies et al., 2007^1^; Bellock et al., 2021), we can compute the convex hull or alpha shape of the coordinates of cell types, and compute the area of these polygons. We can then ask if, on average, these metrics shift across groupings of arrays (e.g., healthy and diseased samples).

The full potential of spatial transcriptomics arises when we look into the spatial distribution of cell types, paired with their gene expression profiles. Searching for spatially non-random distributions of gene expression can be done independent of cell type information, but it is hard to distinguish gene hits that are simply markers for cell types, which themselves are characterized by distinct spatial organization. For this reason, it is useful to look for spatially non-random genes within specific cell types. Several methods currently exist that accomplish this task of discovering spatially non-random genes. In the first Slide-seq paper (Rodriques et al., 2019), the authors presented a permutation-based approach, which compares the distribution of randomly selected beads (while accounting for the total number of transcripts for each bead) with the distribution of beads expressing the specific gene of interest. They also defined spatially overlapping/anticorrelating gene pairs, regional gene enrichment and other interpretable measures using similar permutation based methods, to tackle important biological questions such as quantification and visualization of local transcriptional responses to injury. It is notable, however, that the permutation processes they used were time-consuming and included manual downstream filtering.

Another study introduces SPARK (Sun et al., 2020), which utilizes a generative, generalized linear model, argued to be more statistically robust and computationally efficient than preceding methods. It assumes that gene counts can be modeled by an over dispersed Poisson distribution, where the rate parameter is dictated by a non-spatial component (weighted sum of covariates, including batch information, cell-cycle information etc.), and a spatial component, dictated by either a gaussian distribution (representative of a localized gene expression pattern) or periodic distribution. Each component has a different variance, and the method addresses the null hypothesis that the variance of the spatial component is 0. The SPARK developers argue that a generative statistical model is far more efficient than permutation-based approaches.

Another unique advantage of coupling gene expression data with spatial information is inferring cell interactions. Previously, cell interactions were drawn from scRNA-seq, identifying ligand-receptor co-expression across cell type pairs (Cabello-Aguilar et al., 2020; Lu et al., 2021). However, there was no way of knowing if identified signaling was truly occurring between cell types in close vicinity to each other. With the onset of new spatial transcriptomics technologies, new methods have been developed to increase our confidence in identifying signaling between cell types by accounting for spatial information. Giotto looks for coexpression of ligand-receptor pairs, specifically within cell types in close vicinity to each other (Dries et al., 2021b), while MESSI is a method that uses multi-task learning to predict response genes to intracellular and intercellular signals, considering expression from neighboring cells (Li D. et al., 2021).

## Conclusion

In this manuscript, we have reviewed the biological and computational basis of spatial transcriptomics analysis, with an example of cell type mapping and downstream applications in kidney tissue. Spatial transcriptomics technologies are evolving at a rapid pace and extending beyond transcriptomics into metabolomics and proteomics (Lundberg and Borner, 2019; Ganesh et al., 2021; Yuan et al., 2021). The integration of unbiased spatial omics technologies will provide a powerful set of tools to characterize disease processes in intact tissue (Dries et al., 2021a). We hope that these technologies will not only develop data rich atlases of healthy and diseased tissues, but also provide a platform for advances in the fundamental understanding of disease mechanisms and highlight new therapeutic targets.

## Author Contributions

TN, QW, and JM designed, wrote, and edited the manuscript. JM prepared the figure. AG edited the manuscript. All authors contributed to the article and approved the submitted version.

## Conflict of Interest

The authors declare that the research was conducted in the absence of any commercial or financial relationships that could be construed as a potential conflict of interest.

## Publisher’s Note

All claims expressed in this article are solely those of the authors and do not necessarily represent those of their affiliated organizations, or those of the publisher, the editors and the reviewers. Any product that may be evaluated in this article, or claim that may be made by its manufacturer, is not guaranteed or endorsed by the publisher.

## Footnotes

1 Gillies, S., et al. (2007). Shapely: Manipulation and Analysis of Geometric Objects. Available online at: https://github.com/shapely/shapely/blob/main/CITATION.txt
